# Kolmogorovian versus Non-Kolmogorovian Probabilities in Contextual Theories

**DOI:** 10.3390/e23010121

**Published:** 2021-01-18

**Authors:** Claudio Garola

**Affiliations:** Department of Mathematics and Physics, University of Salento, Via Arnesano, 73100 Lecce, Italy; garola@le.infn.it

**Keywords:** contextuality, non-Kolmogorovian probabilities, quantum probability, quantum measurements

## Abstract

Most scholars maintain that quantum mechanics (QM) is a contextual theory and that quantum probability does not allow for an epistemic (ignorance) interpretation. By inquiring possible connections between contextuality and non-classical probabilities we show that a class $T^{\mu \mathrm{MP}}$ of theories can be selected in which probabilities are introduced as classical averages of Kolmogorovian probabilities over sets of (microscopic) contexts, which endows them with an epistemic interpretation. The conditions characterizing $T^{\mu \mathrm{MP}}$ are compatible with classical mechanics (CM), statistical mechanics (SM), and QM, hence we assume that these theories belong to $T^{\mu \mathrm{MP}}$. In the case of CM and SM, this assumption is irrelevant, as all of the notions introduced in them as members of $T^{\mu \mathrm{MP}}$ reduce to standard notions. In the case of QM, it leads to interpret quantum probability as a derived notion in a Kolmogorovian framework, explains why it is non-Kolmogorovian, and provides it with an epistemic interpretation. These results were anticipated in a previous paper, but they are obtained here in a general framework without referring to individual objects, which shows that they hold, even if only a minimal (statistical) interpretation of QM is adopted in order to avoid the problems following from the standard quantum theory of measurement.

## 1. Introduction

Probability enters quantum mechanics (QM) via Born’s rule and it is usually interpreted in terms of frequencies of the outcomes that are obtained when measurements are performed. However, it turns out to be non-Kolmogorovian, in the sense that the probability measures that are associated with quantum states do not satisfy the assumptions of Kolmogorov’s probability theory. In particular, the set of events of every probability measure that is associated with a quantum state is endowed with the structure of a *standard quantum logic* (QL), which is a non-distributive orthomodular lattice (except for some special cases), at variance with the set of events in Kolmogorov’s theory, which is endowed with a structure of Boolean lattice.

The standard interpretation of QM introduces another nonclassical feature of QM, i.e., the doctrine that, whenever a physical system in a given state is considered, a quantum observable generally has not a prefixed value but only a set of *potential* values, and that a measurement on an individual example of a physical system (*individual object* in the following) actualizes one of these values, yielding an outcome that depends on the specific measurement procedure that is adopted (*contextuality*). This doctrine is usually maintained to be proven correct by some “no-go” theorems that supply a mathematical support to the standard (Copenhagen) interpretation of QM and show, in particular, that contextuality also occurs in the case of measurements on far-away subsystems of a composite physical system (*nonlocality*). However, maintaining that QM deals with individual objects and their properties raises deep problems in the quantum theory of measurement (in particular, the *objectification problem*, see, e.g., [1]), which are considered to be still unsolved by many scholars and imply known paradoxes. There is a huge literature on these topics, which goes back to the early days of QM. Here, we limit ourselves to recall that the EPR and the QL issues were started by the famous papers by Einstein, Podolski, and Rosen [2] and by Birkhoff and von Neumann [3], respectively, while the nonlocality and, more generally, the contextuality of QM were accepted by most physicists as “mathematically proven” after the publication of Bell’s [4,5] and Kochen–Specker’s [6]) theorems (later supported by numerous different proofs of the same or similar theorems, among which, in particular, the proof of nonlocality that was provided by Greenberger, Horne, Shimony, and Zeilinger [7], which does not resort to inequalities).

Because of contextuality, it is a widespread belief that quantum probability does not admit an epistemic interpretation (the term *epistemic* is meant here in a broad sense, i.e., as referring to our degree of knowledge/lack of knowledge). Indeed, one cannot generally consider a property of a quantum system as either possessed or not possessed by the system independently of any measurement, even if the state of the system is known. Hence, one cannot look at the values of the probability measure that are associated with the state as indexes of the degree of ignorance of the properties possessed by the system. Probability should rather be seen as an irreducible feature of the microworld: in this sense, it is *ontic*.

The standard view expounded above is obviously legitimate, but a further investigation on possible links between quantum probability and contextuality may suggest alternative views. We have inquired into such links in a previous paper [8], where macroscopic contexts associated with sets of microscopic contexts (μ*-contexts*) were introduced and quantum probability measures were interpreted as classical averages of Kolmogorovian probability measures on μ-contexts. (A valuable “contextual approach to quantum formalism” has been provided also by Khrennikov [9,10] in the framework of the “Växjö school”. However, this approach is basically different from ours. Khrennikov considers indeed contexts “as a generalization of a widely used notion of *preparation procedure*” [10], which includes also *selection procedures* that are *registration procedures* in the sense of Ludwig [11]. In our approach, instead, macroscopic measurement procedures are associated with macroscopic measurement contexts, which seems to be compatible with Khrennikov’s view, but an essential role is played by the μ-contexts that underlie macroscopic measurement contexts, which are not considered by Khrennikov.) At the best of our knowledge, our approach is innovative, as it focuses on an analysis and rational reconstruction of the basic language of QM, thus adopting a methodology that is typical of analytic philosophy, but is rather unusual in physics. Yet, the language worked out in [8] is a first order predicate calculus, in which an individual variable *x* is interpreted on individual objects. Hence, our results rest on an interpretation of QM that is not free of problems and paradoxes. In the present paper, we propose a more general view, singling out a class of theories, including classical mechanics (CM), statistical mechanics (SM), and QM, in which non-Kolmogorovian probability measures are introduced as derived notions in a Kolmogorovian framework, taking into account contextuality, but making no reference to individual objects. In the case of QM, this means that our results hold, even if one just assumes that the values of the probability measures that are associated with quantum states are the large numbers limits of relative frequencies of measurement outcomes (the *minimal interpretation* of QM). Thus, they are compatible both with a statistical interpretation, according to which no reference to individual systems should be made, and with a “realistic” interpretation, according to which QM deals with individual objects and their properties (see, e.g., [1]). Therefore, let us summarily describe our procedures.

First of all, after some epistemological and physical preliminaries (Section 2 and Section 3, respectively), we consider, in Section 4, a class T of theories in which the basic notions of *physical system* (or *entity*), *state*, *property* and *macroscopic context* are introduced, and then work out a propositional language *L* that, for every T∈T, in which every macroscopic context can be associated with a set of μ-contexts, formalizes a fragment of the natural language expressing basic features of the entities considered in T. The set of elementary (or *atomic*) propositions of *L* is partitioned into a subset of *atomic state propositions* and a subset of *atomic context-depending propositions*. A proposition of the former subset affirms that an entity *H* of T is in a given state. A proposition of the latter subset affirms that an entity *H* of T possesses a given property in a given μ-context (we stress that no atomic proposition assigning a property of *H* without referring to a μ-context exists in *L*). Subsequently, we select a subclass $T^{\mu \mathrm{MP}}\subset T$ by introducing a classical probability measure on the set of all (atomic and molecular) propositions of *L* (Section 5) and a family of classical probability measures, each of which is defined on a subset of μ-contexts (Section 6), and corresponds to a *measurement procedure* that determines a *macroscopic measurement context* and is associated with a property. Thus, we can define a notion of *compatibility* on the set of all properties of *L* and a notion of *testability* on the set of all propositions of *L*, and use the foregoing classical probability measures conjointly to define the notion of *mean conditional probability* on the subset of all testable propositions, together with the related notion of *mean probability test*. Hence, the mean conditional probabilities admit an *epistemic* interpretation, but are not bound to satisfy Kolmogorov’s axioms, because they are obtained by averaging over classical probability measures.

Based on the definitions and results that are expounded above, we focus (Section 7) on the set E of all properties, on which a family of mappings $E⟶\left[0,1\right]$ can be introduced by means of mean conditional probabilities, being parametrized by the set S of all states. This family induces a preorder relation ≺ on E. We show that, if suitable structural conditions are satisfied, then each element of the family is a *generalized probability measure* (or *q-probability*) on (E,≺), which reduces to a classical probability measure whenever (E,≺) is a Boolean lattice. Generalized probability measures can be empirically tested and they admit an epistemic interpretation, but they generally do not satisfy Kolmogorov’s assumptions. Moreover, they allow for the definition of a new kind of conditioning that refers to a sequence of measurements and is conceptually different from classical conditioning.

Thus, we are ready to discuss the implications of our framework in the special cases of CM, SM, and QM. To this end, we first show that the characterizing features of $T^{\mu \mathrm{MP}}$ are compatible with these theories (Section 8); hence, we assume that CM, SM and QM belong to $T^{\mu \mathrm{MP}}$ (there are nowadays also nonphysical theories that can be maintained to belong to $T^{\mu \mathrm{MP}}$, as the models in cognitive sciences that use a quantum formalism, see, e.g., [12,13]), but we do not deal with this issue in the present paper.) This assumption is irrelevant in CM and SM. Indeed, leaving apart SM for the sake of brevity, we prove in Section 9 that all of the notions introduced above collapse into standard notions in CM, consistent with the non-contextual character of this theory. On the contrary, the above assumption has a great explanatory power in QM. Indeed, by considering QM in Section 10, we recover the following results that have been anticipated in [8].

(i) The probability measures on the set of properties that are induced by the Born rule in QM can be considered as the specific form that q-probabilities take in QM. Hence, they are interpreted as derived notions within a Kolmogorovian framework and their non-classical character can be explained in classical terms. This explanation implies that quantum probability can be provided with an epistemic rather than an ontic interpretation by taking into account μ-contexts.

(ii) The relation of compatibility on the set of all physical properties that occurs in QM can be considered to be a specific form of the relation of compatibility that is introduced in our general framework.

(iii) The conditional probability usually introduced in quantum probability can be considered as the specific form of the new kind of conditioning that is introduced in our general framework.

As we have anticipated above, these results are now obtained without referring to individual objects (no individual variable occurs in *L*); hence, they hold, even if only a minimal or a statistical interpretation of QM is accepted that avoids the problems that are raised by the standard quantum theory of measurement.

Finally, we conclude our paper with some summarizing remarks (Section 9), and add an Appendix. This addition is motivated by the fact that the notions of mean conditional probability and mean probability test are conceptually similar to the notions of *universal average* and *universal measurement*, respectively, as introduced by Aerts and Sassoli de Bianchi (see, e.g., [14,15]). In particular, in the case of QM, our approach provides a description of the measurements which test probabilities that recalls the proposal of these authors. Indeed, in our framework, μ-contexts are associated (generally many-to-one) with macroscopic measurement procedures, not with states or properties of the entity that is being measured (hence, our approach is not a hidden variables theory for QM, at least in a standard sense), which reminds Aerts’ hidden measurements approach [16]. However, there are also some relevant differences between the two approaches. In particular, quantum probability is considered to be irreducible, i.e., ontic in the sense established above, by Aerts and Sassoli de Bianchi, while we prove in the present paper that it also admits an epistemic interpretation. Therefore, our Appendix aims to provide a brief and intuitive account of the aforesaid similarities and differences.

To close, we notice that there are several recent attempts in the literature at avoiding the objectification problem by denying the existence of a physical system in the interval between two interactions: see, e.g., Mohrhoff [17], and Licata and Chiatti [18]. In our opinion, it is unclear whether quantum probability can be considered to be epistemic in these approaches. In any case, they introduce interpretations of the formalism of QM that are alternative to the standard interpretation, while our results only require the weak assumptions of the minimal interpretation of QM, which makes them compatible with the standard interpretation. Moreover, also Licata and Chiatti take into account the Aerts et al. approach to QM, hypothesizing a correspondence between the “localizations” that occur in their description of the measurement process and the “hidden measurements” introduced by Aerts [16,19], but they do not go into the details of this correspondence.

## 2. Epistemological Preliminaries

According to the epistemological view called *standard epistemological conception*, or *received view* (see, e.g., [20,21,22,23]), a fully-developed physical theory T is, in principle, expressible by means of a metalanguage in which a *theoretical language* $L_{T}$, an *observational language* $L_{O}$ and *correspondence* (or *epistemic*) *rules* $R_{C}$ connecting $L_{T}$ and $L_{O}$ can be distinguished. The theoretical apparatus of T, expressed by means of $L_{T}$, includes a *mathematical structure* and, usually, an *intended interpretation*, which is a *direct* and *complete* physical model of the mathematical structure (this model is often anticipated by the choice of the nouns of the theoretical terms and it is not indispensable in principle, but it plays a fundamental role in the intuitive comprehension, justification, and development of the theory; think, e.g., to the trajectories of point-like particles in CM or to the geometrical representation of electromagnetic waves). The observational language $L_{O}$ describes instead an empirical domain, hence it has a semantic interpretation, so that the correspondence rules $R_{C}$ provide an *empirical interpretation* of the mathematical structure. However, such an interpretation is often complicated and/or problematic (e.g., one-dimensional orthogonal projection operators may represent both a pure state and a dichotomic observable in QM, i.e., different physical entities). Moreover, it is generally *indirect*, in the sense that there are theoretical entities that are connected with the empirical domain only via *derived* theoretical entities, and *incomplete*, in the sense that only limited ranges of values of the theoretical entities are interpreted (e.g., self-adjoint operators correspond in QM to measuring apparatuses whose outcomes only match the eigenvalues of the operators in finite intervals of the real axis).

The received view has been criticized by some authors (see, e.g., [24,25] ) and it is nowadays maintained to be outdated by several scholars. Nevertheless, we deem that its basic ideas are still epistemologically relevant. In particular, this view led us to focus our attention on the languages of physical theories, and to analyze their syntax and semantics to find out the roots of several open problems in the foundations of such theories. The results that we have obtained are sometimes unexpected and they challenge well established beliefs (see, e.g., [8,26,27,28]).

We add that in the standard language of physical theories the distinctions introduced by the received view are usually overlooked, and the various linguistic components are mixed together (e.g., the term “observable” may denote, in QM, a self-adjoint operator on a Hilbert space H and, in this sense, it belongs to $L_{T}$, but also a physical entity that is associated with a set of measurement procedures and, in this sense, it belongs to $L_{O}$; the term “state” may denote a vector of H, but also a physical entity that is associated with a set of preparing procedures; etc.). Only a rational reconstruction of the language of a theory can lead to clearly distinguishing the various elements that occur in it according to the received view. Therefore, for the sake of simplicity, here we retain some of the basic ideas of such view that we consider epistemologically relevant, but adopt a simpler scheme. To be precise, we maintain that every advanced scientific theory T is expressible by means of a fragment of the natural language that is enriched with technical terms and it is characterized by a pair (F,I), with *F* being a logical and mathematical formalism that may have an intended interpretation and *I* an *empirical interpretation*, indirect and incomplete in the sense explained above, that establishes connections between *F* and an empirical domain. Moreover, in some locutions (as “the minimal interpretation of QM”, etc.), the interpretation *I* is distinguished from the theory, following a standard use.

## 3. Physical Preliminaries

The main ideas for our general treatment are suggested by a typical case of contextual theory, i.e., QM. Therefore, we consider this theory in the present section. For the sake of intuitivity, here we refer to an interpretation of QM that is “realistic” in the sense that it assumes that QM deals with individual objects and their properties (see, e.g., [1]), even if our general theory avoids referring to individual objects, as anticipated in Section 1. Moreover, we adopt a standard physical language in which the distinctions emphasized in Section 2 are not explicitly introduced.

First of all, we recall that, in most presentations of QM, the notions of *physical system*, or *entity*, *(physical) property* and *(physical) state* are fundamental, and that, according to some known approaches to the foundations of QM (see, e.g., [11,29]), states are empirically interpreted as classes of probabilistically equivalent *preparation procedures*, or *preparing devices*, and properties as classes of probabilistically equivalent *dichotomic (yes-no) registering devices*. This interpretation suggests an intuitive explanation of the fact that QM only yields probabilistic predictions. Indeed, one can adopt a picture according to which a microscopic world underlies the macroscopic world of our everyday experience and note that there are two possible sources of randomness for the outcomes of a measurement, as follows (see also [30]).

(i) When an individual object is prepared by activating a preparation procedure that is associated with a state *S*, we only control macroscopic variables, not the physical situation at the microscopic level. Thus, different individual objects that are produced by the preparation procedure are not bound to yield the same outcomes.

(ii) When a registering device is activated to perform a measurement, many *microscopic contexts* can be associated with it, and different microscopic contexts that we cannot control may affect, in different ways, the outcome of the measurement.

However, the picture above does not distinguish QM from SM. This crucial distinction can be established as follows. Consider QM and a preparation procedure π in the class *S*. When activated, π produces an individual object *x* (which can be identified with the act of activation itself if one wants to avoid ontological commitments). Hence, after the activation, a sentence that affirms that *x* is in the state *S* is *true* and a sentence that affirms that *x* is in a state $S^{\prime }\neq S$ is *false*. Subsequently, given an individual object *x* in the state *S*, activating a registering device *r* in the class *E* performs a test of the property *E*, but the result of the test generally depends on the set of properties (pairwise compatible and compatible with *E*) that are tested, together with *E*. In fact, it follows from some known proofs of Bell’s and Kochen–Specker’s theorems (see, e.g., [7,31]) that, if the laws of QM have to be preserved in every conceivable physical situation, the outcome that is obtained depends on the set of the registering devices that are activated together with *r*, i.e., on the macroscopic context $C_{m}$ determined by the whole (macroscopic) measurement *m* that is performed. Briefly, QM is a *contextual* theory, at variance with SM.

Contextuality means that it is impossible in QM to assign a truth value to a sentence stating that *x* has (or *possesses*) a property *E* disregarding the measurement context. In other words, the natural everyday language and the technical language of classical physics, whose elementary sentences state properties of individual objects independent of any observation, are unsuitable for QM (which is the source of most “quantum paradoxes” in our opinion). This fundamental feature of QM was clearly implicit in Bohr’s holistic view (see, e.g., [32]) or in Heisenberg’s distinction between “potential” and “actual” properties (see, e.g., [33]), but it was maintained to be definitively “mathematically proven” only after the statement of Bell’s and Kochen–Specker’s theorems. This notwithstanding, it is strongly counterintuitive, as it implies nonobjectivity of physical properties, Einstein’s "spooky action at a distance" etc. At first sight, one can think that a possible answer to these problems may consist in assuming that the basic language of QM is the nonstandard logic of quantum propositions that was introduced by Birkhoff and von Neumann [3], which implies a nonclassical notion of truth (*quantum truth*) according to some authors (see, e.g., [34,35]). However, this answer does not grasp the point in our opinion. Indeed, we have proven in some previous papers that quantum logic can be embedded (preserving the order, but not the algebraic structure) into a classical logic [26,36] or into a pragmatic extension of classical logic [28]. Moreover, these results are supported by some former results, which show that there are examples of classical macroscopic systems that exhibit a quantum structure (see, e.g., [19]).

On the other side, one can maintain that contextuality, implying a breakdown with a classical view of the world, is a fundamental feature that should be incorporated into the basic language of QM rather than recognized at a later stage. By associating this idea with the above picture of the sources of randomness in QM, we observe that, generally, the macroscopic context $C_{m}$ that is determined by a macroscopic measurement *m* may be produced by many different microscopic physical situations that cannot be distinguished at a macroscopic level (although they can be described, in principle, by QM itself). Hence, we can associate $C_{m}$ with a set $C_{m}$ of *microscopic contexts* (μ*-contexts*; of course, $C_{m}$ could reduce to a singleton in special cases) and then assume that the truth value of a sentence asserting that *x* possesses the property *E* generally depends on *m* through the μ-context that is realized when *m* is performed. However, we cannot know this μ-context; hence, only a probability of it can be given which expresses our degree of ignorance of it (here we naively argue as though the set $C_{m}$ were discrete, in order to avoid technical complications).

Summing up, our picture leads us to conclude that a truth value can be supposed to exist only in the case of a sentence asserting that an individual object *x* possesses a property *E* in a given μ-context *c*, not in the case of a sentence simply asserting that *x* possesses a property *E*. Moreover, in general, this value cannot be deduced from the laws of QM, which are probabilistic laws that make no explicit reference to contexts: hence, we generally do not know it.

The conclusions above have an important consequence. Every quantum prediction concerns probabilities, hence testing it requires evaluating frequencies of outcomes. A typical test of this kind consists in preparing a broad set of individual objects in a given state *S* and then performing on each object the same macroscopic measurement *m* by activating one or more (compatible) registering devices. The macroscopic context $C_{m}$ is then the same for every individual object, but the μ-context $c\in C_{m}$ generally changes in an unpredictable way. Thus, we meet two distinct sources of randomness. The first is the state *S*, be it a pure state or a mixture (see (i) above; note that we could introduce μ-contexts also referring to the preparation procedures that are associated with *S* by the empirical interpretation, but this would uselessly complicate our framework). The second is the unpredictable change of the μ-context that occurs when performing *m* on different individual objects (see (ii) above). Because of the former source, we would generally obtain different results when iterating *m*, even if we could fix the μ-context $c\in C_{m}$, so that for every property *E* we could evaluate a frequency approaching (in the large numbers limit) the probability that an individual object in the state *S* possesses the property *E* in the μ-context *c*. Because of the latter source, we can only assign a probability to every $c\in C_{m}$ and then conclude that the frequencies that are obtained actually approach the mean over $C_{m}$ of the foregoing probabilities. It is then reasonable to identify this mean with the quantum probability of *E* in the state *S*, which implies that quantum probabilities simultaneously take both of the sources of randomness listed above into account. In the next sections, we will see that this idea, together with contextuality, can explain the non-Kolmogorovian character of quantum probabilities, together with the rather surprising fact that their values neither depend on μ-contexts nor on macroscopic contexts (see, e.g., [31]). However, in order to avoid unnecessary restrictions of our framework, we will not refer in the following to individual objects and only consider measurements directly testing probabilities, consistent with the minimal interpretations of QM (see Section 1).

**Remark** **1.** *We have emphasized in some previous papers (see, e.g., [37,38,39,40]) that the epistemological clause “the laws of QM have to be preserved in every conceivable physical situation” is essential in the proofs of Bell’s and Kochen–Specker’s theorems. Indeed all such proofs proceed ab absurdo, as they consider physical situations in which non-compatible physical properties are assumed to be simultaneously possessed by an individual object, and show that this assumption leads to contradictions with well established quantum laws. Nevertheless, the aforesaid clause is generally not explicitly noticed or stated, possibly because it seems to be unquestionably justified by the outstanding success of QM. One can then object that in the physical situations that are considered the quantum laws that are applied can never be simultaneously tested, hence hypothesizing that they hold anyway seems to be more consistent with a classical than with a quantum view. Therefore, one can try to give up the aforesaid clause, but then the proofs of Bell’s and Kochen–Specker’s theorems cannot be completed. This conclusion opens the way to the attempt at recovering noncontextual interpretations of QM (see, e.g., [27,41]). However, we adhere to the standard view in the present paper, even if our arguments apply to every theory in which contexts can be defined, irrespective of whether the results of measurements are context-dependent (locally, or also at a distance) or not.*

## 4. The Classical Propositional Language *L*

We introduce the following definition while bearing in mind the epistemological and physical preliminaries shown in Section 2 and Section 3.

**Definition** **1.** *We denote by T the class of theories in which the notions of entity, property, and state are explicitly introduced, together with a notion of measurement (hence, implicitly, of macroscopic context). We then denote by $T^{\mu }\subset T$ the subclass of theories in which each macroscopic context can be associated with a set of microscopic contexts (μ-contexts).*

We now implement the idea of incorporating contextuality in the basic language of a theory $T\in T^{\mu }$ by constructing a formalized language *L* that is intended to provide a rational reconstruction of the basic language of every $T\in T^{\mu }$ (hence, *L* can be considered as a part of the formalism of T). To this end, we agree to use standard symbols in set theory and logic. In particular, ${}^{c}$, ∩, ∪, ⊂, ∖, ∅, and P(Ψ) will denote complementation, intersection, union, inclusion, difference, empty set, and power set of the set Ψ, respectively. Furthermore, *N* will denote the set of natural numbers, and *iff* the locution "if and only if".

**Definition** **2.** *We call entity the triple H=(E, S, C), where E, S, and C are disjoint sets whose elements we call properties, states and μ-contexts, respectively. Furthermore, we denote by L a classical propositional language, endowed with the following syntax and semantics.*

Syntax.

*(i) A set*$\Pi_{\mathrm{EC}}^{a}=\left\{\alpha_{Ec}|E\in E,c\in C\right\}$*of atomic* context-depending propositions, *a set* $\Pi_{S}^{a}=\left\{\alpha_{S}|S\in S\right\}$ *of atomic* state propositions *and a set* $\Pi^{a}$$=\Pi_{\mathrm{EC}}^{a}\cup \Pi_{S}^{a}$ *of* atomic *propositions.*

*(ii) Connectives* ¬ *(not),* ∧ *(and),* ∨ *(or).*

*(iii) Parentheses* (*,*).

*(iv) A set*Π*of atomic and molecular propositions of L, obtained by applying recursively standard formation rules in classical logic (to be precise, for every*$A\in \Pi^{a}$, A∈Π*; for every*A∈Π, ¬A∈Π*; for every*A,B∈Π*,*A∧B∈Π*and*A∨B∈Π*)*.

Semantics.

*A set W of truth assignments on*Π*, each element of which is a mapping*

w:Π⟶{t,f}

*(where t stands for true and f for false) that satisfies the standard (recursive) assignment rules of classical logic (to be precise, let*A,B∈Π*; then,*w(¬A)=t*iff*w(A)=f*,*w(A∧B)=t*iff*w(A)=t*and*w(B)=t*,*w(A∨B)=t*iff*w(A)=t*or*w(B)=t*) and, furthermore, it is such that, for every S,*$S^{\prime }\in S$, $S\neq S^{\prime }$*implies that*$w(\alpha_{S^{\prime }})=f$, *whenever*$w(\alpha_{S})=t$.

We explicitly note that the last clause in the definition of *w* is suggested by the interpretation of states as equivalence classes of preparation procedures (see Section 3).

The logical preorder and the Lindenbaum–Tarski algebra of *L* can then be introduced in a standard way, as follows.

**Definition** **3.** *We denote, by < and ≡, the (reflexive and transitive) relation of logical preorder and the relation of logical equivalence onΠ, respectively, defined by standard rules in classical logic (to be precise, for every*A,B∈Π*,*A<B*iff, for every*w∈W*,*w(B)=t*whenever*w(A)=t*, and*A≡B*iff*A<B*and*B<A*). Moreover, we put*$\Pi^{\prime }=\Pi /\equiv$*and denote by*${<}^{\prime }$*the partial order canonically induced by < on*$\Pi^{\prime }$*. Hence,*$\left(\Pi^{\prime },{<}^{\prime }\right)$*is a Boolean lattice (the Lindenbaum–Tarski algebra of L) and we denote by*$\neg^{\prime }$*,*$\wedge^{\prime }$*,*$\vee^{\prime }$*the operations canonically induced on*$\Pi^{\prime }$*by* ¬, ∧, ∨*, respectively.*

As stated in Definition 2, the language *L* is a classical propositional language. However, its interpretation introduces some innovative features. Indeed, the words *state*, *property*, and μ-*context* occur in Definition 2 just as nouns of elements of sets, but they obviously refer to an empirical interpretation that makes such elements correspond to empirical entities that are denoted by the same nouns. Moreover, a state *S* is associated in *L* with a state-proposition $\alpha_{S}$ that is an argument of truth assignments and is interpreted as “the entity *H* is in the state *S*” (at variance with known views in quantum logic that consider states as *possible worlds* of a Kripkean semantics; see, e.g., [35]). A property *E* is associated instead with a family ${\left\{\alpha_{Ec}\right\}}_{c\in C}$ of context-depending propositions of *L*, where $\alpha_{Ec}$ is argument of truth assignments and it is interpreted as “the entity *H* possesses the property *E* in the μ-context *c*”.

## 5. A μ-Contextual Probability Structure on *L*

Following Williamson [42], we now introduce a probability measure on *L* by means of the following definitions and propositions.

**Definition** **4.** *Let A∈Π. We set*

Ext:Π⟶P(W),A⟶{w∈W|w(A)=t}

*and say that Ext(A) is the extension of the proposition A.*

We stress that the extension of a proposition *A* generally depends on the μ-contexts that occur in the formal expression of *A*.

**Proposition** **1.** *The mapping Ext satisfies the following conditions.*

*(i) For every A∈Π, $Ext\left(\neg A\right)=W\setminus Ext\left(A\right)={\left(Ext\left(A\right)\right)}^{c}$.*

*(ii) For every A,B∈Π, Ext(A∧B)=Ext(A)∩Ext(B).*

*(iii) For every A,B∈Π, Ext(A∨B)=Ext(A)∪Ext(B).*

*(iv) For every A∈Π, Ext(A∨¬A)=W and* Ext(A∧¬A)=∅ *.*

*(v) For every A,B∈Π, A<B if Ext(A)⊂Ext(B) and A≡B if Ext(A)=Ext(B).*

*Moreover, the algebraic structure $\Theta =(Ext\left(\Pi \right){,}^{c},\cap ,\cup )$ is a Boolean algebra that is isomorphic to $\left(\Pi^{\prime },\neg^{\prime },\wedge^{\prime },\vee^{\prime }\right)$.*

**Proof.**  Straightforward from Definitions 1, 2 and 4. □

Following a standard terminology, we call *classical probability space* here any triple (Ω,Σ,ξ), where Ω is a set, Σ is a Boolean σ-subalgebra of P(Ω), and ξ:Σ⟶[0,1] is a mapping satisfying the following conditions: (i) ξ(Ω)=1; (ii) if ${\left\{\Delta_{i}\right\}}_{i\in N}$ is a family of pairwise disjoint elements of Σ, then $\xi \left(\cup_{i}\Delta_{i}\right)=\Sigma_{i}\xi \left(\Delta i\right)$.

**Definition** **5.** *Let Φ=(W,Θ,ξ) be a classical probability space, let $\Pi^{+}\subset \Pi$ be the set of propositions such that, for every $B\in \Pi^{+}$, ξ(Ext(B))≠0, and let p be a binary mapping such that*

$$p:\Pi \times \Pi^{+}⟶\left[0,1\right],\left(A,B\right)⟶p\left(A|B\right)=\frac{\xi (Ext(A)\cap Ext(B))}{\xi (Ext(B))}.$$

*We say that the pair (Φ,p) is a μ-contextual probability structure on L and that p(A∣B) is the μ-contextual conditional probability of A given B. Moreover, whenever Ext(B)=W we say that p(A∣B) is the μ-contextual absolute probability of A and simply write p(A) in place of p(A∣B).*

The terminology that is introduced in Definition 5 (where the word μ *-contextual* emphasizes that the values of *p* depend on μ-contexts through the propositions of *L*) is justified by the following statement.

**Proposition** **2.** *Let $B\in \Pi^{+}$. The mapping*

$$p_{B}:\Pi ⟶\left[0,1\right],A⟶p\left(A|B\right)$$

*satisfies the following conditions.*

*(i) If A∈Π is such that Ext(A)=W (equivalently, A≡A∨¬A), then $p_{B}\left(A\right)=1$.*

*(ii) If ${\left\{A_{i}\right\}}_{i\in N}$ is a family of propositions such that, for every k,l∈N, $Ext\left(A_{k}\right)\cap Ext\left(A_{l}\right)=\emptyset$ (equivalently, $A_{k}<\neg A_{l}$), then $p_{B}\left(\vee_{i}A_{i}\right)=\sum_{i}p_{B}\left(A_{i}\right)$.*

**Proof.**  Straightforward. □

Proposition 2 shows that, for every $B\in \Pi^{+}$*,* $p_{B}$ is a *probability measure* on (Π,¬,∧,∨). Moreover, it obviously implies Bayes’ theorem, that is the equation p(B)p(A∣B)=p(A)p(B∣A).

**Remark** **2.** *Let R,S∈S and let $\alpha_{S}\in \Pi^{+}$. We obtain from Definition 5*

$$p\left(\alpha_{R}|\alpha_{S}\right)=\frac{\xi (Ext\left(\alpha_{R}\right)\cap Ext\left(\alpha_{S}\right))}{\xi (Ext\left(\alpha_{S}\right))},$$

*which shows that the values of the μ-contextual conditional probability do not always depend on μ-contexts.*

Let us observe now that the above introduction of a probability measure on *L* is purely formal. However, it can be intuitively justified by resorting to the picture of the world provided in Section 3. Indeed, whenever states are interpreted as equivalence classes of preparation procedures, one can generalize the aforesaid picture and assume that activating a preparation procedure π produces an individual object that is in a given state *S* and, for every μ-context *c*, possesses a given set of properties that depend on *c*, thus determining a truth assignment *w* on *L*. Again activating π produces another individual object that still is in the state *S*, but it may possess a different set of properties for some c∈C (indeed we cannot control the preparation procedure at a microscopic level, see (ii) in Section 3), thus determining a truth assignment $w^{\prime }$ on *L* that may differ from *w*. Given a universe U of individual objects, we can then maintain that each individual object can be associated with a truth assignment on *L* and that this correspondence is, generally, many-to-one. Let us roughly reason in finite terms (we are only looking for an intuitive justification of our mathematical structure here) and let us consider the set $Ext(\alpha_{S})$ of all the truth assignments that assign the value *t* (*true*) to the atomic proposition $\alpha_{S}$ stating that the entity *H* is in the state *S* (see Section 4). We can assign a weight to $\alpha_{S}$ that is proportional to the number of individual objects that are associated with truth assignments in $Ext(\alpha_{S})$. Furthermore, similar procedures lead to assigning a weight to the atomic proposition $\alpha_{Ec}$ stating that the entity *H* has the property *E* in the μ-context *c*. Hence, a weight can be assigned to all propositions of *L* following obvious rules. Thus, it is apparent that the Definitions 4 and 5 formalize this idea.

Whenever the above intuitive justification of the μ-contextual probability structure on *L* is accepted, such a structure can be seen as a theoretical expression of the source of randomness that is described in Section 3, (i), and it is important to stress that it is basically classical. Hence, μ-contextual conditional probabilities admit an epistemic interpretation. In other words, they can be considered as indexes of our lack of knowledge of the truth assignments on Π. In the framework of a theory T∈T characterized by the pair (F,I) (see Section 2), it may occur that these probabilities can be evaluated by using the laws of T. However, generally, they cannot be tested. Indeed, a test would require that one may fix a μ-context, while one cannot know the physical situation at a microscopic level, hence the μ-context associated, via *I*, with it. Therefore, μ-contextual probabilities must be considered as theoretical entities that can only be empirically interpreted indirectly (see again Section 2). The next Section is then devoted to discuss this issue in greater detail.

## 6. Measurement Procedures

The predictions of a fully developed scientific theory are usually checked by means of measurements whose theoretical description is part of the theoretical language of the theory. In the present paper, we are interested in the theories, in which μ-contexts and tests of probabilities are introduced. Hence, the formal apparatus of each theory of this kind must include not only a μ-contextual probability structure on *L*, but also a theoretical description of the measurements that correspond to tests of probabilities via the empirical interpretation of the theory (see Section 2). The physical preliminaries shown in Section 3 then provide us again with some important suggestions. Firstly, a measurement may refer to more than one atomic proposition simultaneously. Secondly, the theoretical description must consider a subset of μ-contexts that correspond to the possible microscopic empirical situations that underlie the test of probability. Thirdly, a probability measure must be defined on the foregoing subset of μ-contexts in order to take our limited knowledge of the microscopic empirical situation when a test of probability is performed into account.

Bearing in mind the requirements above, we introduce the definition that follows.

**Definition** **6.** *We denote by $T^{\mu M}$ the subclass of $T^{\mu }$ characterized by the following conditions.*

*(i) For every $T\in T^{\mu M}$, a μ-contextual probability structure on L is defined.*

*(ii) For every $T\in T^{\mu M}$, every E∈E is associated with a set $M_{E}$ of measurement procedures, and every $M\in M_{E}$ determines a macroscopic measurement context $C_{M}$ associated with a classical probability space $\left(C_{M},\Sigma_{M},\nu_{M}\right)$, where $C_{M}$ is a subset of μ-contexts, such that, for every $c\in C_{M}$, $\left\{c\right\}$ belongs to $\Sigma_{M}$.*

*(iii) For every S∈S, $\alpha_{S}\in \Pi^{+}$.*

The theoretical notion of measurement procedure that is introduced in Definition 6 is rather abstract, because we want to avoid any reference to individual objects (see Section 1). However, every quantum measurement of the kind considered in Section 3 is a measurement procedure in the sense defined above.

Now, let us observe that, generally, a test of probability refers to non-atomic (i.e., molecular) propositions, which may require considering several atomic propositions (hence, several states, properties, and μ-contexts) simultaneously, consistently with the first suggestion above. Thus, we are naturally led to introduce the notions of *compatibility*, *testability*, and *joint testability*, as follows.

**Definition** **7.** *Let us consider a theory $T\in T^{\mu M}$ and a non-empty countable set $\left\{E,F,\ldots \right\}\in P\left(E\right)$ of properties of L. We say that E,F,… are compatible (in T) if $M_{E}\cap M_{F}\cap \ldots \neq \emptyset$.*

*Moreover, for every A∈Π, let $E_{A}=\left\{E,F,\ldots \right\}$ be the (finite) set of all the properties that occur (as indexes) in the formal expression of A (together with indexes in C). We say that A is testable (in T) if the following conditions hold.*

*(i) No atomic state proposition occurs in A (hence $E_{A}\neq \emptyset$).*

*(ii) E,F,… are compatible.*

*(iii) E,F,… occur in the formal expression of A together with the same index c, and a measurement procedure $M\in M_{E}\cap M_{F}\cap \ldots$ exists such that $c\in C_{M}$.*

*Furthermore, we denote the set of all testable propositions of *Π* by $\Pi_{\tau }$, and for every $A\in \Pi_{\tau }$ we write A(c) in place of A whenever explicit reference to the μ-context c defined in (iii) must be done.*

*Finally, let $\left\{A,B,\ldots \right\}$ be a non-empty finite set of propositions of $\Pi_{\tau }$. We say that A,B,… are jointly testable (in T) if the proposition A∧B∧… is testable.*

Based on Definition 7, we state the following proposition.

**Proposition** **3.** *Let $T\in T^{\mu M}$. The following statements hold in T.*

*(i) Let us denote by k the binary compatibility relation on E defined by setting*

for every E,F∈E, EkF iff E and F are compatible.

*The relation k is reflexive and symmetric, but, generally, not transitive.*

*(ii) Let E∈E, $M\in M_{E}$ and $c\in C_{M}$. The atomic proposition $\alpha_{Ec}$ belongs to $\Pi_{\tau }$.*

*(iii) Let $A\in \Pi_{\tau }$, $E_{A}=\left\{E,F,\ldots \right\}$, $M\in M_{E}\cap M_{F}\cap \ldots$, $c_{0}\in C_{M}$ and $A=A(c_{0})$. The set $A=\left\{A\left(c\right)|c\in C_{M}\right\}$ is a subset of $\Pi_{\tau }$ (equivalently, for every $c\in C_{M}$, $A\left(c\right)\in \Pi_{\tau }$).*

**Proof.**  Straightforward. □

It remains to understand what one actually checks by means of a test of probability performed by means of a measurement procedure $M\in M_{E}\cap M_{F}\cap \ldots$(to be precise, by means of the empirical measurement procedure corresponding to *M* via an empirical interpretation). Therefore, let us again resort to the intuitive picture that is sketched in Section 3 with reference to QM. Such a picture suggests that, if a measurement is performed of the (compatible) properties E,F,… that occur in a proposition $A\left(c_{0}\right)\in \Pi_{\tau }$ by means of a measurement procedure $M\in M_{E}\cap M_{F}\cap \ldots$, then a μ-context occurs which one cannot control. Hence, one cannot know whether the measurement yields the truth value of $A(c_{0})$ or the truth value of another proposition A(c)∈A. When the measurement is iterated, we obtain frequencies that approach a mean over A (hence, over $C_{M}$), in the large numbers limit, of the μ-contextual conditional probabilities that are defined in Section 5.

We add that we are generally interested in a class of theories, in which all of the tests corresponding to measurement procedures that belong to $M_{E}\cap M_{F}\cap \ldots$ yield the same results, which requires that such procedures be probabilistically equivalent (e.g., the registering devices that are considered in Section 3).

The following definition formalizes the above ideas.

**Definition** **8.** *Let $T\in T^{\mu M}$, let $\Pi_{S}=\left\{A\in \Pi |E_{A}=\emptyset \right\}$ be the set of all propositions, in which no symbol of property occurs (briefly, state-propositions), and let A,* $B\in \Pi_{\tau }\cup \Pi_{S}$ *. We introduce the following averages of* μ *-contextual probabilities.*

*(i) Let A, B be jointly testable,* $E_{A}\cup E_{B}=\left\{E,F,\ldots \right\}$ *,* $M\in M_{E}\cap M_{F}\cap \ldots$ *,* $c_{0}\in C_{M}$ *and* $A=A(c_{0})$ *,* $B=B(c_{0})$ *. Moreover, for every* $c\in C_{M}$ *, let* $B\left(c\right)\in \Pi^{+}$ *. We set*

$$<p\left(A|B\right){>}_{C_{M}}=\sum_{c\in C_{M}}\nu_{M}\left(\left\{c\right\}\right)p\left(A\left(c\right)|B\left(c\right)\right).$$

*(ii) Let*$A\in \Pi_{\tau }$*,*$B\in \Pi_{S}$*,*$E_{A}=\left\{E,F,\ldots \right\}$*,*$M\in M_{E}\cap M_{F}\cap \ldots$*,*$c_{0}\in C_{M}$*and*$A=A(c_{0})$. *Moreover, let*$B\in \Pi^{+}$*. We set*

$$<p\left(A|B\right){>}_{C_{M}}=\sum_{c\in C_{M}}\nu_{M}\left(\left\{c\right\}\right)p\left(A\left(c\right)|B\right).$$

*(iii) Let*$A\in \Pi_{S}$*,*$B\in \Pi_{\tau }$*,*$E_{B}=\left\{E,F,\ldots \right\}$*,*$M\in M_{E}\cap M_{F}\cap \ldots$*,*$c_{0}\in C_{M}$*and*$B=B(c_{0})$. Moreover, let $B\left(c\right)\in \Pi^{+}$*for every*$c\in C_{M}$*. We set*

$$<p\left(A|B\right){>}_{C_{M}}=\sum_{c\in C_{M}}\nu_{M}\left(\left\{c\right\}\right)p\left(A|B\left(c\right)\right)$$

*.*

*(iv) Let* $A,B\in \Pi_{S}$ *and* $B\in \Pi^{+}$ *. For every measurement procedure M we set*

$$<p\left(A|B\right){>}_{C_{M}}=\sum_{ccC_{M}}\nu_{M}\left(\left\{c\right\}\right)p\left(A|B\right)=p\left(A|B\right).$$

*In case (iv), the reference to*$C_{M}$*in*$<p\left(A|B\right){>}_{C_{M}}$*can be dropped. Moreover, we denote, by*$T^{\mu \mathrm{MP}}$*, the subclass of*$T^{\mu M}$*that consists of all theories satisfying the condition that, if*$A,B\in \Pi_{\tau }\cup \Pi_{S}$*are such that*$E_{A}\cup E_{B}\neq \emptyset$*, and*$<p\left(A|B\right){>}_{C_{M}}$*is defined, then for every*$N\in M_{E}\cap M_{F}\cap \ldots$*the average*$<p\left(A|B\right){>}_{C_{N}}$*is also defined and coincides with*$<p\left(A|B\right){>}_{C_{M}}$*. Therefore, whenever*$T\in T^{\mu \mathrm{MP}}$*we drop the reference to*$C_{M}$*in*$<p\left(A|B\right){>}_{C_{M}}$*, say that*<p(A∣B)>*is the* mean conditional probability *of A given B and briefly write* <p(A)> *in place of* <p(A∣B)> *if* Ext(B)=W*.*

By referring to Definition 8, we can maintain that, for every $T\in T^{\mu \mathrm{MP}}$, the empirical interpretation makes *M* correspond to a *mean probability test* that produces an outcome which is expected to coincide with <p(A∣B)> in the large numbers limit.

We again stress that mean conditional probabilities are introduced in a Kolmogorovian framework in order to take two different kinds of ignorance into account. First, the lack of knowledge regarding the truth assignments on Π mentioned at the end of Section 5. Second, the ignorance of the μ-contexts to be associated with a probability test. Hence, the mean conditional probabilities admit an *epistemic* interpretation. Notwithstanding this, they are not bound to satisfy Kolmogorov’s assumptions, for they are average quantities. In particular, it follows, from Definition 8, that <p(B)><p(A∣B)> is generally different from <p(A)><p(B∣A)>. Hence, a formal analogous of the Bayes theorem does not hold in the case of mean conditional probabilities.

Finally, let us observe that the above definition of mean conditional probabilities and mean probability tests are conceptually similar to the universal averages and universal measurements, respectively, which were introduced by Aerts and Sassoli de Bianchi [14,15]. Moreover, the recognition that two kinds of lack of knowledge occur when a measurement is performed recalls the perspective that was proposed by these authors. As we have anticipated in Section 1, we therefore make a brief comparison of our approach with Aerts and Sassoli de Bianchi’s in the Appendix A.

## 7. Quantum-Like Probability Measures

The set E of all properties is fundamental in every $T\in T^{\mu \mathrm{MP}}$. We intend to focus on it in the present section and show that the notions and definitions in Section 6 allow us to define, whenever some conditions on mean conditional probabilities are satisfied, a family of quantum-like probability measures on E parametrized by the set of all states. To reach our aim, let us preliminarily recall that, for all E∈E, $M\in M_{E}$ and $c\in C_{M}$, $\alpha_{Ec}$ belongs to $\Pi_{\tau }$ because of Proposition 3, (ii). Subsequently, we introduce the following definition.

**Definition** **9.** *Let us consider a theory $T\in T^{\mu \mathrm{MP}}$, let E∈E, $M\in M_{E}$, $c\in C_{M}$, S∈S, and let $P_{S}\left(E\right)$ be the mean conditional probability of $\alpha_{Ec}$ given $\alpha_{S}$, that is,*

$$\begin{array}{cc} P_{S}\left(E\right)= & <p\left(\alpha_{Ec}|\alpha_{S}\right)>=\sum_{c\in C_{M}}\nu_{M}\left(\left\{c\right\}\right)p\left(\alpha_{Ec}|\alpha_{S}\right)\\ & =\sum_{c\in C_{M}}\nu_{M}\left(\left\{c\right\}\right)\frac{\xi (Ext\left(\alpha_{Ec}\right)\cap Ext\left(\alpha_{S}\right))}{\xi (Ext\left(\alpha_{S}\right))}. \end{array}$$

*We denote by ≺ and ≈ the preorder and the equivalence relation on E, respectively, defined by setting, for every E,F∈E,*

$$E≺F\;iff,\;for\;every\;S\in S,\;P_{S}\left(E\right)\leq P_{S}\left(F\right)$$

*and*

E≈F iff E≺F and F≺E.

It is now important to consider a special case that allows for us to place physical theories, such as CM, SM, and QM, within the general framework constructed in Section 4, Section 5 and Section 6. To this end, we introduce the following definition.

**Definition** **10.** *Let us consider a theory $T\in T^{\mu \mathrm{MP}}$, such that ≺ is a partial order and (E,≺) is an orthocomplemented lattice. We denote meet, join, orthocomplementation, least element and greatest element of (E,≺) by* ⋒*,* ⋓*, ${}^{⊥}$, O and U, respectively. Moreover, we denote by* ⊥ *the (binary) orthogonality relation canonically induced by ${}^{⊥}$ on $\left(E,⋒,⋓{,}^{⊥}\right)$ (we recall that ${}^{⊥}$ is a unary operation on (E,≺) such that, for every E,F∈E, $E^{⊥⊥}=E$, E≺F implies $F^{⊥}≺E^{⊥}$, $E⋒E^{⊥}=O$ and $E⋓E^{⊥}=U$, while* ⊥ *is the non-reflexive and symmetric binary relation on E defined by setting, for every E,F∈E, E⊥F if $E≺F^{⊥}$). Afterwards, for every S∈S, we say that the mapping*

$$P_{S}:E⟶\left[0,1\right],E⟶P_{S}\left(E\right)=<p\left(\alpha_{Ec}|\alpha_{S}\right)>$$

*is a generalized probability measure on $\left(E,⋒,⋓{,}^{⊥}\right)$ iff it satisfies the following conditions.*

*(i) $P_{S}\left(U\right)=1$.*

*(ii) If ${\left\{E_{i}\right\}}_{i\in N}$ is a family of properties that are pairwise disjoint (i.e., for every k,l∈N, $E_{k}⊥E_{l}$), then*

$$P_{S}\left({⋓}_{i}E_{i}\right)=\sum_{i}P_{S}\left(E_{i}\right).$$

*Let E∈E. Whenever $P_{S}$ is a generalized probability measure on $\left(E,⋒,⋓{,}^{⊥}\right)$, we say that $P_{S}\left(E\right)$ is the q-probability of E given S.*

Definition 10 implies that a generalized probability measure $P_{S}$ is a classical probability measure iff $\left(E,⋒,⋓{,}^{⊥}\right)$ is a Boolean lattice. Hence, generally, $P_{S}$ does not satisfy Kolmogorov’s assumptions. Nevertheless, the q-probability $P_{S}\left(E\right)$ of a property E∈E given *S* admits an epistemic interpretation and can be empirically tested, as it is a special case of the mean conditional probability that is introduced in Definition 8. It is then natural to wonder whether a *conditional q-probability* of a property E∈E given another property F∈E can be defined by means of $P_{S}$, generalizing standard procedures in classical propositional logic. However, if one tries to put

$$P_{S}\left(E|F\right)=\frac{P_{S}\left(E⋒F\right)}{P_{S}\left(F\right)},$$

then the mapping

$$P_{SF}:E\in E⟶P_{S}\left(E|F\right)\in \left[0,1\right]$$

is not a generalized probability measure on $\left(E,⋒,⋓{,}^{⊥}\right)$ whenever this lattice is not Boolean. Indeed, consider a property $E=E_{1}⋓E_{2}$, with $E_{1},E_{2}\in E$ and $E_{1}⊥E_{2}$. We obtain

$$P_{SF}\left(E\right)=P_{SF}\left(E_{1}⋓E_{2}\right)=P_{S}\left(E_{1}⋓E_{2}|F\right)=\frac{P_{S}\left(\left(E_{1}⋓E_{2}\right)⋒F\right)}{P_{S}\left(F\right)},$$

that is generally different from

$$\frac{P_{S}\left(\left(E_{1}⋒F\right)⋓\left(E_{2}⋒F\right)\right)}{P_{S}\left(F\right)}=P_{S}\left(E_{1}|F\right)+P_{S}\left(E_{2}|F\right)=P_{SF}\left(E_{1}\right)+P_{SF}\left(E_{2}\right)$$

whenever $\left(E,⋒,⋓{,}^{⊥}\right)$ is not distributive.

However, one can introduce a non-standard kind of conditional probability by considering mean probability tests that are performed in sequence rather than conjointly. Indeed, one can again draw inspiration from QM and single out theories in $T^{\mu \mathrm{MP}}$ where measurement procedures exist that correspond, via empirical interpretation, to mean probability tests that filter the sample of the entity that is considered in a prefixed way, producing a new sample on which the same or a different test can be performed. Moreover, still inspired by QM, we are interested in those mean probability tests that yield frequency 1 when repeated on the new sample. Therefore, we introduce the following definition.

**Definition** **11.** *Let us consider a theory $T\in T^{\mu \mathrm{MP}}$ and for every F∈E let us put $S_{F}=\left\{S\in S|P_{S}\left(F\right)\neq 0\right\}$. We say that a measurement procedure $M\in M_{F}$ is of first kind iff it is associated with a mapping*

$$t_{F}:S_{F}⟶S_{F},S⟶t_{F}\left(S\right)$$

*such that* $P_{t_{F}\left(S\right)}\left(F\right)=1$ *. For every* E∈E *and first kind measurement procedure* $M\in M_{F}$ *we then put*

$$P_{S}\left(E∥F\right)=P_{t_{F}\left(S\right)}\left(E\right).$$

*Moreover, let (E,≺) be an orhocomplemented lattice in T and let $P_{S}$ and $P_{t_{F}\left(S\right)}$ be generalized probability measures on (E,≺). We say that $P_{S}\left(E∥F\right)$ is the conditional q-probability of E given F and S.*

If a theory $T\in T^{\mu \mathrm{MP}}$ contains a first kind measurement procedure $M\in M_{F}$, $S\in S_{F}$, and $P_{S}\left(E∥F\right)$ is defined, then $P_{S}\left(E∥F\right)$ can be tested, as mean probability tests always exist for $P_{t_{F}\left(S\right)}\left(E\right)$ (see Section 6; however, no analogous of the Bayes theorem can be stated for conditional q-probabilities). Definition 11 thus introduces a non-standard conditional probability on (E,≺) that can be tested and coexists with the μ-contextual conditional probability introduced in Definition 5, which instead cannot be tested directly and has the status of a purely theoretical notion.

## 8. Physical Theories

The mathematical apparatus worked out in the previous sections has been contrived bearing in mind CM, SM and QM. Indeed, the language of these theories contains terms denoting entities, properties, states and measurements, hence CM, SM and QM belong to T (see Definition 1). Moreover, microscopic contexts can be introduced in CM, SM and QM as special cases of physical systems, hence these theories belong to $T^{\mu }$ (ib.). By referring then to Definition 6, we see that conditions (i), (ii) and (iii) characterizing $T^{\mu M}$ are compatible with (even if not implied by) CM, SM and QM (condition (iii), in particular, establishes that no “useless” state, i.e., a state *S* such that $p(\alpha_{S})=0$, occurs in a theory $T\in T^{\mu M}$). Finally, the condition characterizing $T^{\mu \mathrm{MP}}$ in Definition 8 states that, for every $T\in T^{\mu \mathrm{MP}}$, the mean conditional probability of a proposition *A* given a proposition *B* does not depend on the choice of the measurement procedure, which also is compatible with CM, SM and QM.

Based on the above arguments, we can now assume that CM, SM and QM belong to $T^{\mu \mathrm{MP}}$. It is then easy to see that all the notions introduced in the previous sections collapse into standard notions in the case of CM and SM. In the case of QM, instead, our assumption explains some relevant aspects of this theory and of quantum probability in terms of the general notions introduced in $T^{\mu \mathrm{MP}}$. We therefore discuss these issues in the following sections, referring to CM and QM only and leaving apart SM, which can be dealt with by extending our treatment of CM in an obvious way.

## 9. Classical Mechanics

Let us begin by listing some basic features of CM, some of which can be deduced at once from the phase space representation of states and properties.

(i) CM deals with individual objects, their (pure) states and (physical) properties. Both macroscopic and microscopic measurement contexts can be introduced in it and supplied with an intuitive (intended) interpretation, but it is assumed that each individual object either possesses or does not possess any property that is considered, independently of any measurement procedure.

(ii) Whenever the state *S* of an individual object *x* is given, the set of all properties possessed by *x* is determined by *S*, and it is different from the set of properties possessed by another individual object in a state $S^{\prime }$ different from *S*.

(iii) For every finite set $\left\{E,F,\ldots \right\}$ of properties and individual object *x*, one can check (at least in principle) which properties in $\left\{E,F,\ldots \right\}$ are possessed by *x* and which are not by performing an (exact) measurement that consists in measuring simultaneously E,F,….

(iv) Different properties can be assumed to have different phase space representations, hence they are not equivalent, in the sense that there are individual objects that possess one of them and not the other.

(v) Every negation of a proposition stating a property is a proposition stating a property, and every (finite) conjunction or disjunction of propositions stating properties is a proposition stating a property (see, e.g., [26]).

(vi) For every property *E* and individual object *x*, a measurement exists (at least as a limit of real measurements) which establishes whether *x* possesses or does not possess the property *E* without perturbing the state *S* of *x*.

Let us now discuss how the general notions introduced in Section 4, Section 5, Section 6 and Section 7 specialize in the case of CM.

First of all, (i) implies that macroscopic and microscopic contexts play no role in the truth assignments on Π. Hence, for every w∈W, E∈E*,* c,d∈C, the equality $w\left(\alpha_{Ec}\right)=w\left(\alpha_{Ed}\right)$ holds in CM, which implies $\alpha_{Ec}\equiv \alpha_{Ed}$ (see Definition 2), $Ext\left(\alpha_{Ec}\right)=Ext\left(\alpha_{Ed}\right)$ (see Definition 4) and $\xi \left(Ext\left(\alpha_{Ec}\right)\right)=\xi \left(Ext\left(\alpha_{Ed}\right)\right)$ (see Definition 5). Therefore, we can drop any reference to μ-contexts in the following. In particular, we write $\alpha_{E}$ and $\Pi_{E}^{a}$ in place of $\alpha_{Ec}$ and $\Pi_{\mathrm{EC}}^{a}$, respectively, and notice that the mapping $\tau :E⟶\Pi_{E}^{a},E⟶\alpha_{E}$ is bijective.

Secondly, (ii) implies that, for every w∈W and S∈S*,* the requirement $w(\alpha_{S})=t$ determines the values of *w* on all (atomic and molecular) propositions of Π: in particular, $w(\alpha_{S^{\prime }})=f$ for every $S^{\prime }\neq S$. Hence $Ext(\alpha_{S})$ (see Definition 4) is a singleton, whose unique element we denote by $w_{S}$. It follows from Definition 5 that, for every E∈E*,* $p\left(\alpha_{E}|\alpha_{S}\right)\in \left\{0,1\right\}$. Moreover, every truth assignment on Π refers to individual objects in a state *S*, hence for every w∈W a state S∈S exists such that $w=w_{S}$. Therefore, the mapping $s:S⟶W,S⟶w_{S}$ is bijective.

Thirdly, let us come to measurements. Then, (iii) implies that, for every countable set $\left\{E,F,\ldots \right\}\in P\left(E\right)$, the properties E,F,… are compatible in the sense established in Definition 7. Moreover, every proposition A∈Π, such that no atomic state proposition occurs in it, is testable, that is, $A\in \Pi_{\tau }$, and for every non-empty finite set $\left\{A,B,\ldots \right\}\in P\left(\Pi_{\tau }\right)$, the propositions A,B,… are jointly testable and the result of an exact measurement neither depends on the macroscopic context nor on the μ-contexts. Therefore, for every $A,B\in \Pi_{\tau }\cup \Pi_{S}$*,* if $B\in \Pi^{+}$ the mean conditional probability <p(A∣B)> is defined (see Definition 8) and coincides with p(A∣B). Thus, the notion of mean conditional probability reduces to the notion of conditional probability. Moreover, the mapping $P_{S}$ introduced in Definition 10 is such that, for every E∈E and S∈S,

$$P_{S}\left(E\right)=p\left(\alpha_{E}|\alpha_{S}\right)\in \left\{0,1\right\}.$$

The results obtained above imply that, for every E,F,…∈E, E≺F (see Definition 9) if $\alpha_{E}<\alpha_{F}$ (see Definition 3). Indeed, let us recall that we have assumed in Definition 6 that, for every S∈S, $\alpha_{S}\in \Pi^{+}$, hence $\xi (Ext\left(\alpha_{S}\right))\neq 0$, which implies $\xi (\left\{w_{S}\right\})\neq 0$ in CM. Therefore, for every E,F∈E, the following sequence of coimplications holds.

$$(E≺F)\;\mathrm{iff}\;(\mathrm{for}\;\mathrm{every}\;S\in S,p\left(\alpha_{E}|\alpha_{S}\right)\leq p\left(\alpha_{F}|\alpha_{S}\right))\;\mathrm{iff}\;(\mathrm{for}\;\mathrm{every}\;S\in S,\;\xi \left(Ext\left(\alpha_{E}\right)\cap Ext\left(\alpha_{S}\right)\right)\leq \xi (Ext\left(\alpha_{F}\right)\cap Ext\left(\alpha_{S}\right))\;\mathrm{iff}\;(\mathrm{for}\;\mathrm{every}\;w_{S}\in W,\;\xi \left(Ext\left(\alpha_{E}\right)\cap \left\{w_{S}\right\}\right)\leq \xi \left(Ext\left(\alpha_{F}\right)\cap \left\{w_{S}\right\}\right))\;\mathrm{iff}\;(Ext\left(\alpha_{E}\right)\subset Ext\left(\alpha_{F}\right))\;\mathrm{iff}\;(\alpha_{E}<\alpha_{F}).$$

It follows that the order structures (E,≺) and $\left(\Pi_{E}^{a},<\right)$ are isomorphic. Moreover, < and ≺ are partial orders. Indeed, (iv) implies that, for every E,F∈E, if E≠F then there is a truth assignment $w_{S}$ which assigns the value *t* to one of the propositions $\alpha_{E}$ and $\alpha_{F}$, and value *f* to the other. Hence $\alpha_{E}\equiv \alpha_{F}$ if $\alpha_{E}=\alpha_{F}$, which implies that < is a partial order on $\Pi_{E}^{a}$. Because of the aforesaid isomorphism, also ≺ is a partial order.

Let us now consider q-probability. Because of (v), $\left(\Pi_{E}^{a},<\right)$ is a Boolean lattice, hence (E,≺) is a Boolean lattice (whose meet, join, and complementation we denote, by abuse of language, by ∩, ∪ and ${}^{c}$, respectively). Thus, for every S∈S, the q-probability $P_{S}$ is a classical probability measure on $\left(E,\cap ,\cup {,}^{c}\right)$ (the proof of this statement follows at once from Proposition 2, because of the equality and isomorphism above). Therefore, for every E,F∈E, the conditional probability in the state *S* of *E* given *F* can be defined in a standard way, as follows

$$P_{S}\left(E|F\right)=\frac{P_{S}\left(E\cap F\right)}{P_{S}\left(F\right)}\in \left\{0,1\right\}$$

(where $P_{S}\left(F\right)\neq 0$, hence $P_{S}\left(F\right)=1$).

Finally, let us consider the conditional q-probability $P_{S}\left(E∥F\right)$. Because of (vi), a first kind measurement procedure exists for every F∈E such that $t_{F}$ (see Definition 11) is the identity mapping. We obtain in this case that $P_{S}\left(E∥F\right)=P_{t_{F}\left(S\right)}\left(E\right)=P_{S}\left(E\right)$. Because $P_{S}\left(E\right)\in \left\{0,1\right\}$, it is easy to see that $P_{S}\left(E\right)=P_{S}\left(E|F\right)$. However, notwithstanding this equality, there is a deep conceptual difference between standard conditional probability and conditional q-probability.

## 10. Quantum Mechanics

In Section 8 we have assumed that CM, SM, and QM belong to $T^{\mu \mathrm{MP}}$. Yet, at variance with CM and SM, QM is a theory in which (macroscopic) contexts play a fundamental role. Whenever *L* is assumed to be the basic language of QM, contextuality implies that the inequality $w\left(\alpha_{Ec}\right)\neq w\left(\alpha_{Ed}\right)$ (which implies $Ext\left(\alpha_{Ec}\right)\neq Ext\left(\alpha_{Ed}\right)$) holds in *L* for some w∈W, E∈E*,* c,d∈C.

Let us consider now Hibert space QM (HSQM). Within HSQM, each entity (physical system) is associated with a Hilbert space H, and each state *S* is represented by a density operator $\rho_{S}$ on H and each property *E* is represented by an orthogonal projection operator $P_{E}$ on H. Because the set of all orthogonal projection operators on H is an orthomodular lattice in which a partial order is defined independently of any probability measure; this representation induces on E an order, which we denote by ≪, and (E,≪) is an orthomodular lattice (the standard quantum logic mentioned in Section 1). Moreover, Born’s rule associates a probability value $Tr\left[\rho_{S}P_{E}\right]$ (that does not depend on any context) with every pair (E,S), hence a quantum probability

$$Q_{S}:E⟶\left[0,1\right],E⟶Tr\left[\rho_{S}P_{E}\right]$$

is defined, which is said to be a *generalized probability measure* on (E,≪) (see, e.g., [29]), and the family ${\left\{Q_{S}\right\}}_{S\in S}$ is *ordering* on (E,≪) (ib.), which means that the order induced by it on E coincides with ≪. Therefore, the lattice structure of (E,≪) can be seen as being induced by ${\left\{Q_{S}\right\}}_{S\in S}$. This feature of HSQM implies that the order ≪ and the probability $Q_{S}$ can be considered as the specific forms that the order ≺ and the mapping $P_{S}$, respectively, take in QM (see Definitions 9 and 10). Thus, we obtain, in QM

$$P_{S}\left(E\right)=<p\left(\alpha_{EC}|\alpha_{S}\right)>=Q_{S}\left(E\right)=Tr\left[\rho_{S}P_{E}\right].$$

Furthermore, if the quantum probability $Q_{S}$ replaces $P_{S}$ in the conditions (i) and (ii) stated in Definition 10, then these conditions are satisfied, which makes the above classification of $Q_{S}$ as generalized probability measure consistent with Definition 10. Thus, we obtain an interpretation of quantum probability measures that leads to considering them mean conditional probabilities. Therefore, they can be seen as derived notions within a Kolmogorovian framework, as we have anticipated in Section 1, which explains their non-classical character, but shows that they admit an epistemic interpretation, at variance with their standard ontic interpretation (see Section 6).

In addition, let us recall that a compatibility relation κ is introduced in QM on the set of all properties by setting, for every pair (E,F) of properties, EκF iff $\left[P_{E},P_{F}\right]=0$. This relation is reflexive and symmetric, but not transitive. Hence, it can be considered as the specific form that the relation *k* introduced in Proposition 3 takes in QM.

Coming to measurements, let us recall that first kind measurement procedures exist in QM (see, e.g., [29,43]) and that the Lüders rule states that, whenever an (ideal) first kind measurement of a property *E* is performed on an ensemble that is described by $\rho_{S}$, the subensemble that passes the measurement is described by the density operator $\frac{P_{E}\rho_{S}P_{E}}{Tr\left[\rho_{S}P_{E}\right]}$. Hence, if we denote by $D\left(H\right)$ the set of all density operators on H, then the mapping

$$\tau_{E}:D\left(H\right)⟶D\left(H\right),\rho_{S}⟶\frac{P_{E}\rho_{S}P_{E}}{Tr\left[\rho_{S}P_{E}\right]}$$

can be considered as the specific form that the mapping $t_{E}$ introduced in Definition 11 takes in QM.

Finally, we recall that the conditional probability $Q_{S}\left(F|E\right)$, in a state *S*, of a property *F* given a property *E*, is defined in QM by referring to a measurement of *F* after a measurement of *E* on an ensemble that is described by $\rho_{S}$, and it is given by $\frac{Tr\left[P_{F}P_{E}\rho_{S}P_{E}P_{F}\right]}{Tr\left[P_{E}\rho_{S}P_{E}\right]}$. Hence, this quantity can be considered as the specific form that the conditional q-probability of *F* given *E* and *S* introduced in Definition 11 takes in QM. Thus, we obtain

$$P_{S}\left(F∥E\right)=Q_{S}\left(F|E\right)=\frac{Tr\left[P_{F}P_{E}\rho_{S}P_{E}P_{F}\right]}{Tr\left[P_{E}\rho_{S}P_{E}\right]}.$$

## 11. Conclusions

A class $T^{\mu \mathrm{MP}}$ of scientific theories can be singled out, in which mean probabilities that do not satisfy the assumptions of Kolmogorov’s probability theory may occur within a Kolmogorovian probabilistic framework because of contextuality, according to the perspective presented in this paper. The conditions characterizing $T^{\mu \mathrm{MP}}$ are compatible with CM, SM, and QM, which therefore can be maintained to belong to this class of theories. In the case of QM, this membership implies that quantum probability measures can be seen as mean conditional probabilities that have a non-classical structure, but admit an epistemic interpretation, which challenges the standard ontic interpretation of quantum probability. In addition, we also obtain that some typical features of QM, as the compatibility relation on the set of all physical properties and the quantum notion of conditional probability, are special cases of general notions that are introduced in $T^{\mu \mathrm{MP}}$. These results are obtained without referring to individual objects, which makes them hold, even if only a minimal interpretation of QM is accepted to avoid the problems of the standard quantum theory of measurement.

## Appendix A

As anticipated in Section 1 and Section 6, we intend to make here a brief comparison of our approach to quantum probability with Aerts and Sassoli de Bianchi’s solution to the measurement problem of QM. This solution was expounded, in particular, in a technical paper [14] and in a book aiming to make it understandable to a wider audience [15]. Our comparison will be made mainly referring to that book, which will make our description of the similarities and differences between the two approaches simple and intuitive.

To begin with, let us recall that the proposal of Aerts and Sassoli de Bianchi finds its roots in Aerts’ *hidden measurements* idea (see, e.g., [16]). By developing this idea Aerts and Sassoli de Bianchi provide a detailed description of the measurement process in QM by constructing an elaborate model whose core is the Bloch representation of the pure states of a spin $\frac{1}{2}$ physical system. This representation, in which every pure state corresponds to a point on the surface of a three-dimensional sphere, is extended by Aerts and Sassoli de Bianchi by considering the points within the Bloch sphere as representative of genuine new states rather than statistical mixtures, as it would occur instead in the standard formalism of QM. The final action of an instrument measuring the spin of the physical system is then represented in the sphere by means of an elastic band connecting the north with the south pole of the sphere. When the measurement is performed, the state of the system moves orthogonally onto the elastic band and sticks to it. Then, the elastic band breaks in a point whose position on the band is unpredictable, leading the state either on the north pole (*spin up*) or on the south pole (*spin down*), depending on the position of the breaking point.

To make quantitative the above qualitative description of the measurement process, Aerts and Sassoli de Bianchi assume that the elastic band is characterized by a probability distribution whose value in a given point of the band is interpreted as the density of probability of breaking at that point. Moreover, when the measurement is repeated, the properties of the new elastic band may be different from the properties of the old one, and in this case the new band is characterized by a different probability distribution. Hence, when the measurement is repeated many times on spin $\frac{1}{2}$ systems in a given state, to predict the frequency of each possible outcome one must average over all probability distributions. The authors call this average *universal average*, and then call the measurement *universal measurement*. A quantum measurement of the spin of the physical system is then assumed to be a measurement of this kind.

The following remarks are now important.

(i) The experimenter can choose to perform a measurement but cannot choose the breaking point of the elastic band, as he takes every possible precaution to avoid influencing the outcome. The breaking point of the band is instead assumed by Aerts and Sassoli de Bianchi to be the result of nondeterministic and unpredictable environmental fluctuations. Hence the elastic band corresponds to a *potentiality region* and physical quantities do not pre-exist to the measurement but are *actualized* by it. Therefore the authors consider quantum probability as ontic (in the sense established in Section 1), which fits in well with the standard interpretation of QM.

(ii) In the description of the measuring process probability occurs twice. Firstly, when the elastic band is characterized by a probability distribution. Secondly, when averaging over probability distributions to obtain a universal average, which intuitively means that every possible distribution has the same probability to occur every time the measurement is repeated (because of (i) the experimenter does not influence in any way the “emerging” of a specific distribution).

(iii) Aerts and Sassoli de Bianchi’s description of spin $\frac{1}{2}$ measurements is not a hidden-variables theory in a standard sense. Indeed, it explains the randomness of the observed outcomes as a consequence of fluctuations in the measuring system, consistently with Aerts’ idea of hidden measurements mentioned above, rather than a consequence of our incomplete knowledge of the real state of the measured entity.

After constructing the above model, Aerts and Sassoli de Bianchi make a considerable effort to generalize it to physical entities whose measurements can give more than two possible outcomes. In this case the mathematical apparatus becomes much more complicated (in particular, the Bloch sphere becomes a hypersphere in a space with more than three dimensions and the elastic band is substituted by a hypermembrane). Nevertheless the basic features of spin $\frac{1}{2}$ measurements pointed out above remain unchanged, hence we will refer to them in the following without entering the details of the general model.

Let us come to our approach. Here a canonical distinction between preparing and registering devices is introduced (Section 3) but no explicit model for the measurement process is proposed. Rather, a very simple picture assuming the existence of a microscopic world underlying the macroscopic world of our everyday experience is provided to intuitively justify our formalism (Section 3 and Section 5). According to this picture, we do not know what is going on at a microscopic level, neither in preparing nor in registering devices. To deal with the first kind of lack of knowledge, we introduce a probability measure on the language *L* of the theories that we are considering, which corresponds to the assignments of probability distributions on elastic bands in Aerts and Sassoli de Bianchi’s description. Yet, our probability measure is introduced because the quantum description of the state of a physical system is maintained to be incomplete, according to the spirit of standard hidden variables theories (but only context-depending propositions occur in *L*, which implies that the “no go” theorems mentioned in Section 1 do not apply): hence, it is considered *epistemic*. To deal with the second kind of lack of knowledge we introduce μ-contexts, which complies with the hidden measurements idea and parallels the introduction of different elastic bands when the measurment is repeated in Aerts and Sassoli de Bianchi’s description. Mean conditional probabilities then parallel universal averages. We, however, do not introduce any assumption of equiprobability (see (ii) above), which makes our approach slightly more general. More important, mean conditional probabilities bear an epistemic interpretation, for they are classical weighted means of epistemic probabilities, at variance with Aerts and Sassoli de Bianchi universal averages.

To close, let us recall that Aerts also introduced *state property systems* (see [19] and related bibliography), which successively evolved in the *state-context-property* (SCoP) formalism (see, e.g., [44]; such a formalism was mainly used for working out a theory of concepts, in particular in the field of quantum cognition). Then, the SCoP formalism can be (partially) translated into the formalism developed in the present paper, and conversely. Indeed, its basic structure can be summarized as follows.

(i) Fundamental notions: *entity*, *state*, *(measurement) context*, *property* (hence the SCoP formalism characterizes a class of theories that also belong to T, see Definition 1).

(ii) Fundamental definitions: set of states Σ, set of contexts *M*, set of properties L; entity (Σ,M,L,μ,ν), where μ:Σ×M×Σ⟶[0,1],(p,e,q)⟶μ(p,e,q) is a *state-transition probability function* that represents the likelihood to transition from the state *p* to the state *q* under the influence of the context *e*, and ν:Σ×L⟶[0,1],(p,a)⟶ν(p,a) is a *property-applicability function* that estimates how applicable is the property *a* to the state *p* of the entity.

Then, based on the physical interpretation of the SCoP formalism, the following bijective correspondences with the formalism introduced in the present paper can be established.

$c_{1}:\Sigma ⟶S,p⟶S$,

$c_{2}:M⟶\cup_{E\in E}M_{E},e⟶M_{e}\in M_{E_{e}}$ for an $E_{e}\in E$.

$c_{3}:L⟶E,a⟶E_{a}$.

Moreover, μ(p,e,q) has no equivalent in our framework: nevertheless, if $M_{e}$ is a first kind measurement, then *q* can be identified with $t_{E_{e}}\left(S\right)$ and μ(p,e,q) with $P_{S}\left(E_{e}\right).$

Finally, $c_{1}$ and $c_{3}$ imply that ν(p,a) can be identified with $P_{S}\left(E_{a}\right)$.

By using the correspondences above, the aforementioned (partial) translation can be obtained, which shows that also in this case there are strong structural similarities between Aerts’ approach and ours.
